# Molecular Control of TiO_2_-NPs Toxicity Formation at Predicted Environmental Relevant Concentrations by Mn-SODs Proteins

**DOI:** 10.1371/journal.pone.0044688

**Published:** 2012-09-04

**Authors:** Yinxia Li, Wei Wang, Qiuli Wu, Yiping Li, Meng Tang, Boping Ye, Dayong Wang

**Affiliations:** 1 Key Laboratory of Developmental Genes and Human Disease in Ministry of Education, Department of Biochemistry and Molecular Biology, Medical School of Southeast University, Nanjing, China; 2 College of Life Science and Technology, China Pharmaceutical University, Nanjing, China; 3 School of Public Health, Southeast University, Nanjing, China; "Mario Negri" Institute for Pharmacological Research, Italy

## Abstract

With growing concerns of the safety of nanotechnology, the *in vivo* toxicity of nanoparticles (NPs) at environmental relevant concentrations has drawn increasing attentions. We investigated the possible molecular mechanisms of titanium nanoparticles (Ti-NPs) in the induction of toxicity at predicted environmental relevant concentrations. In nematodes, small sizes (4 nm and 10 nm) of TiO_2_-NPs induced more severe toxicities than large sizes (60 nm and 90 nm) of TiO_2_-NPs on animals using lethality, growth, reproduction, locomotion behavior, intestinal autofluorescence, and reactive oxygen species (ROS) production as endpoints. Locomotion behaviors could be significantly decreased by exposure to 4-nm and 10-nm TiO_2_-NPs at concentration of 1 ng/L in nematodes. Among genes required for the control of oxidative stress, only the expression patterns of *sod-2* and *sod-3* genes encoding Mn-SODs in animals exposed to small sizes of TiO_2_-NPs were significantly different from those in animals exposed to large sizes of TiO_2_-NPs. *sod-2* and *sod-3* gene expressions were closely correlated with lethality, growth, reproduction, locomotion behavior, intestinal autofluorescence, and ROS production in TiO_2_-NPs-exposed animals. Ectopically expression of human and nematode Mn-SODs genes effectively prevented the induction of ROS production and the development of toxicity of TiO_2_-NPs. Therefore, the altered expression patterns of Mn-SODs may explain the toxicity formation for different sizes of TiO_2_-NPs at predicted environmental relevant concentrations. In addition, we demonstrated here a strategy to investigate the toxicological effects of exposure to NPs upon humans by generating transgenic strains in nematodes for specific human genes.

## Introduction

The potential toxicity of materials at the “nano” scale is receiving increasing attentions [1]–[2]. Titanium dioxide nanoparticles (TiO_2_-NPs) are often used as pigments or additives for paints, paper, ceramics, plastics, foods, and other products [3]–[4]. Hence, TiO_2_-NPs come into close contact with humans. The adverse effects caused by exposure to TiO_2_-NPs *in vivo* seem to have become more important than those due to exposure *in vitro*. Acute exposure to TiO_2_-NPs has been shown to cause hepatic injury, nephrotoxicity, pathological changes in the kidney, myocardial damage, spleen lesions, and inflammation in the lung and liver in mice or rats [5]–[10]. After intraperitoneal injection of TiO_2_-NPs, mice showed signs of toxicity, including passive behavior, loss of appetite, tremors and lethargy [7]. It was reported that mice exposed to TiO_2_-NPs during gestation produced offspring that could display moderate neurobehavioral alterations [10]. After subchronic dermal exposure of TiO_2_-NPs, TiO_2_-NPs penetrated through the skin to reach different tissues and induced the formation of diverse pathological lesions in several major organs [11]. In mice, exposure to TiO_2_-NPs also induced fetal resorption, restricted the growth of fetuses, and altered the expression of genes related to development and function of the central nervous system in pregnant animals, indicating the potential fetotoxicity of TiO_2_-NPs [12]–[13]. Acute toxicity assay of TiO_2_-NPs using *Daphnia magna* demonstrated that mortality increased after exposure [14]. Long-term exposure to TiO_2_-NPs has been shown to disturb progression of the cell cycle and to duplicate genome segregation, leading to chromosomal instability and cell transformation [15]. Chronic exposure to TiO_2_-NPs resulted in the inhibition of growth, a decrease in the liver weight ratio, and histopathological changes in the gills in zebrafish [16].

With regard to the biological effects mediated by NPs, evidence was provided to support the notion that NPs-mediated cellular response was size-dependent, which may provide important insights on our understanding of nanotoxicity [17]. In mice exposed to subchronic concentrations of TiO_2_-NPs through the skin, significant decreases in body weight were observed after exposure to TiO_2_-NPs of diameter 10 nm, 25 nm and 21 nm (P25), whereas no obvious differences in body weight were found after exposure to TiO_2_-NPs of diameter 60 nm, and it is unclear the expression and order of 10 nm, 25 nm, and 21 nm (P25) TiO_2_-NPs [11]. In zebrafish, the acute toxicity of TiO_2_-NPs was greater than that of bulk TiO_2_ [18]. In *Caenorhabditis elegans*, acute exposure to TiO_2_-NPs of diameter 25 nm increased mortality, whereas acute exposure to 100-nm TiO_2_-NPs elicited no obvious effects in nematodes [19]. TiO_2_-NPs of diameter 7 nm seemed to be more toxic than 20-nm TiO_2_-NPs in nematodes [20]. Nevertheless, the molecular mechanisms underpinning the formation of size-dependent toxicity for TiO_2_-NPs are largely unclear.

The adverse effects of NPs exposure include (at the very least) oxidative stress, mitochondrial perturbation, inflammation, protein denaturation, protein degradation, altered regulation of the cell cycle and DNA damage [2]. For the development of toxicity of TiO_2_-NPs, oxidative stress may be induced by deposited TiO_2_-NPs. This is because the assay on levels of superoxide dismutase (SOD) and malondialdehyde (MDA) in mice and TiO_2_-NPs enhanced the production of reactive oxygen species (ROS) in cultured fibroblast cells [11], [15]. Oxidative stress was shown to develop in the brain, liver and gut tissues of rainbow trout or zebrafish as indicated by biomarkers of oxidative stress [18], [21]. However, the molecular basis for oxidative stress in the induction of TiO_2_-NPs toxicity is largely unknown.

*C. elegans* is a free-living nematode found mainly in the liquid phase of soils. It is one of the most thoroughly studied model animals. It has been developed in various environmental studies to address the toxicological effects of a wide range of toxicants from the molecular to individual level [22]. Lethal and sublethal endpoints, including development, reproduction, metabolism, and locomotion behavior can be used for environmental toxicological studies [23]–[36]. Recently, *C. elegans* has been used for the evaluation of the toxicity of Zn-NPs, Al-NPs, Ag-NPs, Si-NPs, Ce-NPs, TiO_2_-NPs, metallofullerenol and quantum dots [20], [30]–[31], [34]–[44]. It has been reported that acute exposure to TiO_2_-NPs at relatively high concentrations causes an increase in the mortality, inhibition of growth, reduction of reproduction, and alteration of gene expression patterns in *C. elegans* [20], [37]. However, the role of oxidative stress in the formation of the toxicity differences in nematodes exposed to TiO_2_-NPs at different nanolevels is unclear. In addition, the possible adverse effects of TiO_2_-NPs at predicted environmental relevant concentrations in nematodes are not known. In *C. elegans*, oxidative stress is controlled mainly by the genes of: *sod-1*, *sod-2*, *sod-3*, *sod-4*, and *sod-5* encoding five SODs; *ctl-1*, *ctl-2*, and *ctl-3* encoding three catalases; *clk-1* and *clk-2* encoding a ubiquinone biosynthetic enzyme and a homolog of yeast Tel2p; *isp-1* encoding the “Rieske” iron-sulfur protein; *gas-1* encoding a subunit of mitochondrial complex I required for oxidative phosphorylation; and *mev-1* encoding a subunit of the enzyme succinate dehydrogenase cytochrome b [45]–[51].

In the present study, we investigated the molecular basis of oxidative stress in inducing the differences in toxicity for TiO_2_-NPs of different sizes using *C. elegans* as the assay system. Our data suggested that Mn-SODs proteins may contribute to the formation of different toxicities for different sizes of TiO_2_-NPs at predicted environmental relevant concentrations.

## Results

### Adverse effects of different sizes of TiO_2_-NPs on survival, growth and reproduction of nematodes

**Figure 1:**
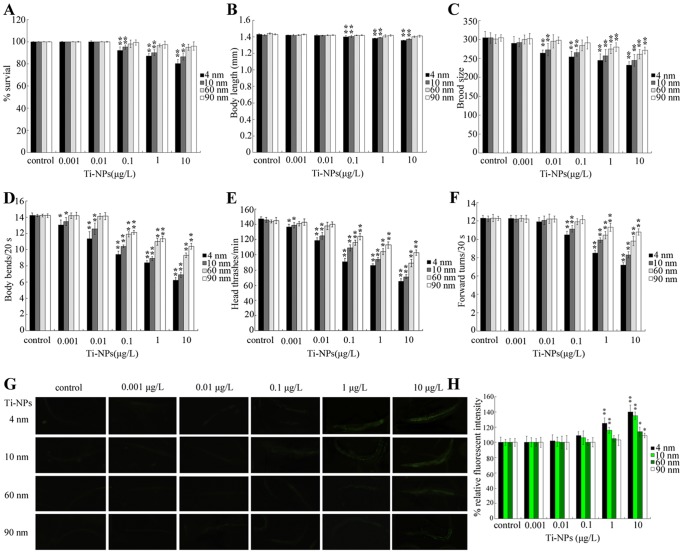
Effects of different sizes of TiO_2_-NPs on survival, growth, reproduction, locomotion behavior and intestinal autofluorescence of nematodes. (A) Effects of different sizes of TiO_2_-NPs on survival of nematodes. (B) Effects of different sizes of TiO_2_-NPs on growth of nematodes. (C) Effects of different sizes of Ti-NPs on reproduction of nematodes. (D–F) Effects of different sizes of TiO_2_-NPs on locomotion behavior of nematodes. (G–H) Comparison of intestinal autofluorescence in nematodes exposed to different sizes of TiO_2_-NPs. Exposure of TiO_2_-NPs was performed from L1-larvae, and the endpoints were examined when nematodes developed into the adults. Ti-NPs, TiO_2_-NPs. The Bars represent mean±S.E.M. *p<0.05, **p<0.01.

Previous study demonstrated that *C. elegans* can be employed to assess the toxicity of nanomaterials at predicted environmental relevant concentrations by exposing *C. elegans* from L1-larvae to adults [42]. The effects of exposure to TiO_2_-NPs at predicated environmental relevant concentrations upon nematodes are unclear. Hence, we first investigated the possible adverse effects of exposure to TiO_2_-NPs at predicated environmental relevant concentrations upon the survival, growth, and reproduction of nematodes by exposing *C. elegans* from L1-larvae to adults. Exposure to 60-nm and 90-nm TiO_2_-NPs at concentrations from 0.001 µg/L to 10 µg/L, and exposure to 4-nm and 10-nm TiO_2_-NPs at concentrations from 0.001 and 0.01 µg/L did not obviously influence the survival of nematodes (Fig. 1A). In contrast, exposure to 4-nm and 10-nm TiO_2_-NPs at concentrations from 0.1 µg/L to 10 µg/L significantly (*p*<0.01) increased the mortality of nematodes (Fig. 1A).

The endpoint of growth was relatively less sensitive than the endpoint of lethality when assessing the toxicity of exposure to TiO_2_-NPs at predicated environmental relevant concentrations. Exposure to all the examined sizes of TiO_2_-NPs at concentrations of 0.001–0.1 µg/L did not obviously influence the growth of nematodes as reflected by body length (Fig. 1B). Exposure to 60-nm and 90-nm TiO_2_-NPs at concentrations of 1–10 µg/L also did not noticeably affect the growth of nematodes (Fig. 1B). However, the body lengths of nematodes exposed to 1 and 10 µg/L of 4-nm or 10-nm TiO_2_-NPs were significantly (*p*<0.01) decreased compared with those of control nematodes (Fig. 1B).

We further examined the possible effects of TiO_2_-NPs exposure at predicated environmental relevant concentrations on the reproduction of nematodes using brood size as the endpoint. Exposure to 0.001 µg/L of all the examined TiO_2_-NPs, as well as exposure to 60-nm and 90-nm TiO_2_-NPs at 0.01 and 0.1 µg/L, did not cause alterations in the brood size of nematodes (Fig. 1C). In contrast, exposure to 4-nm and 10-nm TiO_2_-NPs at 0.01 and 0.1 µg/L significantly (*p*<0.01) reduced the brood size of nematodes (Fig. 1C). Exposure to all the examined TiO_2_-NPs at 1 and 10 µg/L also significantly (*p*<0.01) decreased the brood size of nematodes (Fig. 1C). These data implied that reproduction was relatively more sensitive than lethality and growth if assessing the toxicity of TiO_2_-NPs in nematodes.

### Adverse effects of different sizes of TiO_2_-NPs on locomotion behavior of nematodes

Locomotion behavior is a relatively sensitive endpoint if evaluating the toxicity of a specific toxicant in *C. elegans* [52]. Next, we investigated the effects of TiO_2_-NPs exposure at predicated environmentally relevant concentrations on locomotion behavior by evaluating the body bend, head thrash, and forward turn of nematodes. Exposure to 60-nm and 90-nm TiO_2_-NPs at 0.001 and 0.01 µg/L did not obviously influence the body bends and head thrashes of nematodes (Figs 1D and 1E). In contrast, exposure to 4-nm and 10-nm TiO_2_-NPs at 0.001 µg/L moderately but significantly (*p*<0.05) suppressed the body bends and head thrashes of nematodes, and exposure to 4-nm and 10-nm TiO_2_-NPs at 0.01 µg/L significantly (*p*<0.01) decreased the body bends and head thrashes of nematodes (Figs 1D and 1E). Moreover, exposure to all the examined TiO_2_-NPs at 0.1–10 µg/L significantly (*p*<0.01) reduced the frequencies of body bends and head thrashes of nematodes (Figs 1D and 1E). Different from the observations on body bend and head thrash, forward turn was less sensitive than body bend and head thrash if assessing the toxicity of TiO_2_-NPs in nematodes. Exposure to all the examined TiO_2_-NPs at 0.001–0.01 µg/L and exposure to 60-nm and 90-nm TiO_2_-NPs at 0.1 µg/L did not noticeably affect forward turns (Fig. 1F). Significantly (*p*<0.01) reduced forward turns were observed in nematodes exposed to 4-nm and 10-nm TiO_2_-NPs at 0.1–10 µg/L as well as in those exposed to 60-nm and 90-nm TiO_2_-NPs at 1–10 µg/L (Fig. 1F).

### Effects of different sizes of TiO_2_-NPs on intestinal autofluorescence of nematodes

Intestinal autofluorescence is a valuable marker of damage in intestinal cells in nematodes [53]. Our data suggested that exposure to all the examined TiO_2_-NPs at 0.001–0.1 µg/L as well as 60-nm and 90-nm TiO_2_-NPs at 1 µg/L did not induce noticeable increases in intestinal autofluorescence compared with those in controls (Figs 1G and 1H). In contrast, exposure to 4-nm and 10-nm TiO_2_-NPs at 1–10 µg/L significantly (*p*<0.01) induced an increase in intestinal autofluorescence compared with controls (Figs 1G and 1H). In addition, exposure to 60-nm and 90-nm TiO_2_-NPs at 10 µg/L moderately but significantly (*p*<0.05) increased intestinal autofluorescence compared with those seen in controls (Figs 1G and 1H). These data implied that the possible obvious damage in intestinal cells was formed only in nematodes exposed to relatively high concentrations of TiO_2_-NPs.

### Effects of different sizes of TiO_2_-NPs on ROS production of nematodes

**Figure 2:**
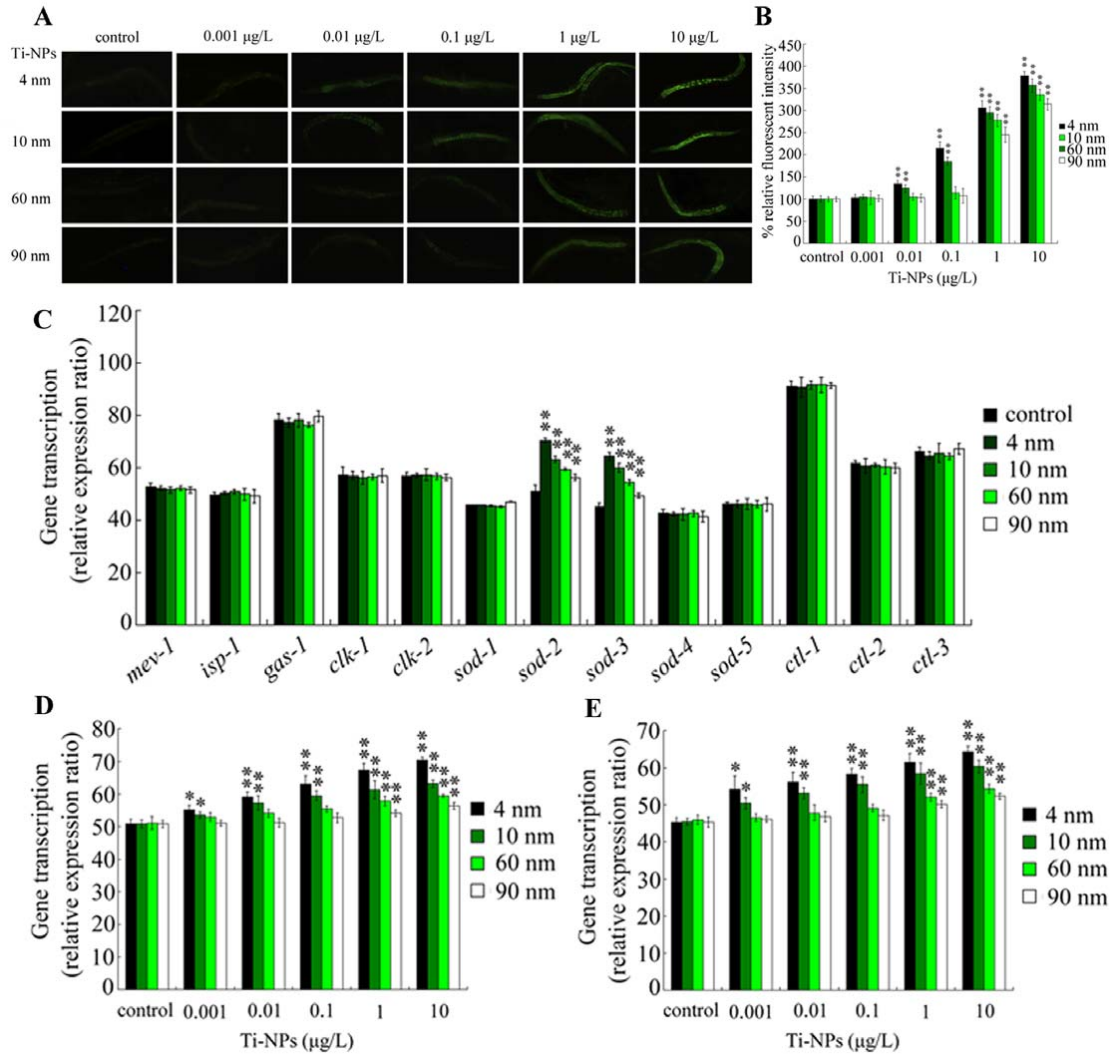
Effects of different sizes of TiO_2_-NPs on ROS production and expression patterns of genes controlling oxidative stress in nematodes. (A–B) Comparison of ROS production in nematodes exposed to different sizes of TiO_2_-NPs. (C) Expression patterns of genes controlling oxidative stress in nematodes exposed to different sizes of TiO_2_-NPs at the concentration of 10 µg/L. (D) Expression patterns of *sod-2* gene in nematodes exposed to different sizes of TiO_2_-NPs. (E) Expression patterns of *sod-3* gene in nematodes exposed to different sizes of TiO_2_-NPs. Relative expression ratios (between target genes and *act-1* reference gene) in treatments were normalized to the control. Exposure of TiO_2_-NPs was performed from L1-larvae, and the endpoints were examined when nematodes developed into the adults. Ti-NPs, TiO_2_-NPs. The Bars represent mean±S.E.M. *p<0.05, **p<0.01.

We further investigated the possible effects of TiO_2_-NPs exposure at predicated environmental relevant concentrations on the ROS production of nematodes. Interestingly, although no obvious induction was observed in nematodes exposed to all the examined TiO_2_-NPs at 0.001 µg/L, significant (*p*<0.01) induction of ROS production was detected in nematodes exposed to 4-nm and 10-nm TiO_2_-NPs at 0.01–10 µg/L compared with those in controls (Figs 2A and 2B). In contrast, only exposure to 60-nm and 90-nm TiO_2_-NPs at 1–10 µg/L activated a significant (*p*<0.01) increase in ROS production in nematodes compared with those observed in controls (Figs 2A and 2B).

### Associations of ROS production with lethality, growth, reproduction, locomotion behavior and intestinal autofluorescence in nematodes exposed to TiO_2_-NPs

Linear regression analyses were undertaken to examine the possible associations of ROS production with lethality, growth, reproduction, locomotion behavior and intestinal autofluorescence in nematodes exposed to different sizes of TiO_2_-NPs. The dependent variables were lethality, growth, reproduction, locomotion behavior and intestinal autofluorescence, and the independent variable was ROS production. Under our experimental conditions, ROS production was significantly correlated with lethality (R^2^ = 0.985, *p*<0.01), growth (R^2^ = 0.986, *p*<0.01), reproduction (R^2^ = 0.828, *p*<0.05), body bend (R^2^ = 0.935, *p*<0.01), head thrash (R^2^ = 0.916, *p*<0.01), forward turn (R^2^ = 0.996, *p*<0.01), and intestinal autofluorescence (R^2^ = 0.967, *p*<0.01) in nematodes exposed to 4-nm TiO_2_-NPs (Table S1). ROS production was significantly correlated with lethality (R^2^ = 0.992, *p*<0.01), growth (R^2^ = 0.985, *p*<0.01), reproduction (R^2^ = 0.809, *p*<0.05), body bend (R^2^ = 0.960, *p*<0.01), head thrash (R^2^ = 0.949, *p*<0.01), forward turn (R^2^ = 0.978, *p*<0.01), and intestinal autofluorescence (R^2^ = 0.918, *p*<0.01) in nematodes exposed to 10-nm TiO_2_-NPs (Table S1). ROS production was significantly correlated with lethality (R^2^ = 0.944, *p*<0.01), growth (R^2^ = 0.724, *p*<0.05), reproduction (R^2^ = 0.868, *p*<0.01), body bend (R^2^ = 0.837, *p*<0.05), head thrash (R^2^ = 0.839, *p*<0.05), forward turn (R^2^ = 0.992, *p*<0.01), and intestinal autofluorescence (R^2^ = 0.878, *p*<0.01) in nematodes exposed to 60-nm TiO_2_-NPs (Table S1). ROS production was significantly correlated with lethality (R^2^ = 0.981, *p*<0.01), growth (R^2^ = 0.719, *p*<0.05), reproduction (R^2^ = 0.900, *p*<0.01), body bend (R^2^ = 0.795, *p*<0.05), head thrash (R^2^ = 0.837, *p*<0.05), forward turn (R^2^ = 0.997, *p*<0.01), and intestinal autofluorescence (R^2^ = 0.904, *p*<0.01) in nematodes exposed to 90-nm TiO_2_-NPs (Table S1). Therefore, ROS production was significantly correlated with lethality, growth, reproduction, locomotion behavior, and intestinal autofluorescence in nematodes exposed to all the examined TiO_2_-NPs at predicated environmentally relevant concentrations.

### Exposure to TiO_2_-NPs alters the expression patterns of genes encoding Mn-SODs

In *C. elegans*, oxidative stress is controlled by key genes such as *sod-1*, *sod-2*, *sod-3*, *sod-4*, *sod-5, ctl-1*, *ctl-2*, *ctl-3, clk-1, clk-2, isp-1, gas-1,* and *mev-1* [45]–[51]. To examine the possible molecular basis of oxidative stress in the induction of toxicity differences in nematodes exposed to different diameters of TiO_2_-NPs, we investigated the expression patterns of key genes controlling the oxidative stress in nematodes exposed to different sizes of TiO_2_-NPs at 10 µg/L. Among the 13 genes examined, only the expression patterns of *sod-2* and *sod-3* genes were noticeably altered (Fig. 2C). Expression levels of the *sod-2* gene and *sod-3* gene were significantly (*p*<0.01) increased after exposure to 4-nm, 10-nm, 60-nm, or 90-nm TiO_2_-NPs at 10 µg/L compared with those observed in controls. The expression patterns of *sod-2* and *sod-3* genes encoding Mn-SODs in nematodes exposed to 4-nm and 10-nm TiO_2_-NPs were also different from those in nematodes exposed to 60-nm and 90-nm TiO_2_-NPs (Fig. 2C). Moreover, with the increases of exposure concentrations of different diameters of TiO_2_-NPs, the expression levels of *sod-2* or *sod-3* genes increased gradually compared with those of controls (Figs 2D and 2E). In nematodes, expressions of *sod-2* and *sod-3* genes were significantly increased by exposure to 4-nm and 10-nm TiO_2_-NPs at 0.001–10 µg/L as well as exposure to 60-nm and 90-nm TiO_2_-NPs at 1–10 µg/L (Figs 2D and 2E). These data suggested that genes encoding Mn-SODs may account for the development of toxicity differences in nematodes exposed to different sizes of TiO_2_-NPs.

The involvement of *sod-2* and *sod-3* genes in the control of oxidative stress was analyzed further by linear regression analyses on the associations of *sod-2* and *sod-3* gene expression with ROS production. The dependent variable was ROS production, and the independent variables were *sod-2* and *sod-3* gene expressions. Under our experimental conditions, *sod-2* gene expression was significantly correlated with ROS production in nematodes exposed to 4-nm (R^2^ = 0.914, *p*<0.01), 10-nm (R^2^ = 0.805, *p*<0.05), 60 nm (R^2^ = 0.823, *p*<0.05) and 90-nm (R^2^ = 0.913, *p*<0.01) TiO_2_-NPs (Table S2). *sod-3* gene expression was also significantly correlated with ROS production in nematodes exposed to 4-nm (R^2^ = 0.745, *p*<0.05), 10-nm (R^2^ = 0.807, *p*<0.05), 60-nm (R^2^ = 0.915, *p*<0.01) and 90-nm (R^2^ = 0.956, *p*<0.01) TiO_2_-NPs (Table S2). Similarly, *sod-2* or *sod-3* gene expression was positively correlated with ROS production in nematodes exposed to 4-nm, 10-nm, 60 nm, and 90-nm TiO_2_-NPs as assayed by Spearman's rank correlation (Table S3).

### Associations of *sod-2* and *sod-3* gene expression with lethality, growth, reproduction, locomotion behavior and intestinal autofluorescence in nematodes exposed to Ti-NPs

Linear regression analyses were carried out to examine the possible associations of *sod-2* and *sod-3* gene expression with lethality, growth, reproduction, locomotion behavior and intestinal autofluorescence in nematodes exposed to different sizes of TiO_2_-NPs. The dependent variables were lethality, growth, reproduction, locomotion behavior and intestinal autofluorescence, and the independent variables were *sod-2* and *sod-3* gene expressions. *sod-2* gene expression was significantly correlated with lethality (R^2^ = 0.860, *p*<0.01), growth (R^2^ = 0.921, *p*<0.01), reproduction (R^2^ = 0.973, *p*<0.01), body bend (R^2^ = 0.985, *p*<0.01), head thrash (R^2^ = 0.973, *p*<0.01), forward turn (R^2^ = 0.887, *p*<0.01), and intestinal autofluorescence (R^2^ = 0.823, *p*<0.05) in nematodes exposed to 4-nm TiO_2_-NPs (Table S2). *sod-2* gene expression was significantly correlated with lethality (R^2^ = 0.772, *p*<0.05), growth (R^2^ = 0.810, *p*<0.05), reproduction (R^2^ = 0.997, *p*<0.01), body bend (R^2^ = 0.914, *p*<0.01), head thrash (R^2^ = 0.928, *p*<0.01), forward turn (R^2^ = 0.753, *p*<0.05), and intestinal autofluorescence (R^2^ = 0.662, *p*<0.051) in nematodes exposed to 10-nm TiO_2_-NPs (Table S2). *sod-2* gene expression was significantly correlated with lethality (R^2^ = 0.872, *p*<0.01), growth (R^2^ = 0.890, *p*<0.01), reproduction (R^2^ = 0.944, *p*<0.01), body bend (R^2^ = 0.864, *p*<0.05), head thrash (R^2^ = 0.859, *p*<0.01), forward turn (R^2^ = 0.696, *p*<0.05), and intestinal autofluorescence (R^2^ = 0.681, *p*<0.05) in nematodes exposed to 60-nm TiO_2_-NPs (Table S2). *sod-2* gene expression was significantly correlated with lethality (R^2^ = 0.966, *p*<0.01), growth (R^2^ = 0.874, *p*<0.01), reproduction (R^2^ = 0.982, *p*<0.01), body bend (R^2^ = 0.944, *p*<0.01), head thrash (R^2^ = 0.965, *p*<0.01), forward turn (R^2^ = 0.940, *p*<0.01), and intestinal autofluorescence (R^2^ = 0.872, *p*<0.01) in nematodes exposed to 90-nm TiO_2_-NPs (Table S2). Similarly, *sod-3* gene expression was significantly correlated with lethality (R^2^ = 0.684, *p*<0.05), growth (R^2^ = 0.797, *p*<0.05), reproduction (R^2^ = 0.918, *p*<0.01), body bend (R^2^ = 0.885, *p*<0.01), head thrash (R^2^ = 0.859, *p*<0.01), forward turn (R^2^ = 0.716, *p*<0.05), and intestinal autofluorescence (R^2^ = 0.664, *p*<0.05) in nematodes exposed to 4-nm TiO_2_-NPs (Table S2). *sod-3* gene expression was significantly correlated with lethality (R^2^ = 0.702, *p*<0.05), growth (R^2^ = 0.807, *p*<0.05), reproduction (R^2^ = 0.978, *p*<0.01), body bend (R^2^ = 0.905, *p*<0.01), head thrash (R^2^ = 0.914, *p*<0.01), forward turn (R^2^ = 0.754, *p*<0.05), and intestinal autofluorescence (R^2^ = 0.668, *p*<0.05) in nematodes exposed to 10-nm TiO_2_-NPs (Table S2). *sod-3* gene expression was significantly correlated with lethality (R^2^ = 0.959, *p*<0.01), growth (R^2^ = 0.786, *p*<0.05), reproduction (R^2^ = 0.985, *p*<0.01), body bend (R^2^ = 0.948, *p*<0.01), head thrash (R^2^ = 0.966, *p*<0.01), forward turn (R^2^ = 0.952, *p*<0.01), and intestinal autofluorescence (R^2^ = 0.820, *p*<0.05) in nematodes exposed to 60-nm TiO_2_-NPs (Table S2). *sod-3* gene expression was significantly correlated with lethality (R^2^ = 0.970, *p*<0.01), growth (R^2^ = 0.871, *p*<0.01), reproduction (R^2^ = 0.973, *p*<0.01), body bend (R^2^ = 0.867, *p*<0.01), head thrash (R^2^ = 0.918, *p*<0.01), forward turn (R^2^ = 0.975, *p*<0.01), and intestinal autofluorescence (R^2^ = 0.875, *p*<0.01) in nematodes exposed to 90-nm TiO_2_-NPs (Table S2). Therefore, *sod-2* or *sod-3* gene expression was significantly correlated with lethality, growth, reproduction, locomotion behavior, and intestinal autofluorescence in nematodes exposed to all the examined TiO_2_-NPs at predicted environmental relevant concentrations.

### Ectopically expression of nematode *sod-2* or *sod-3* prevents the toxicity formation in nematodes exposed to TiO_2_-NPs

**Figure 3:**
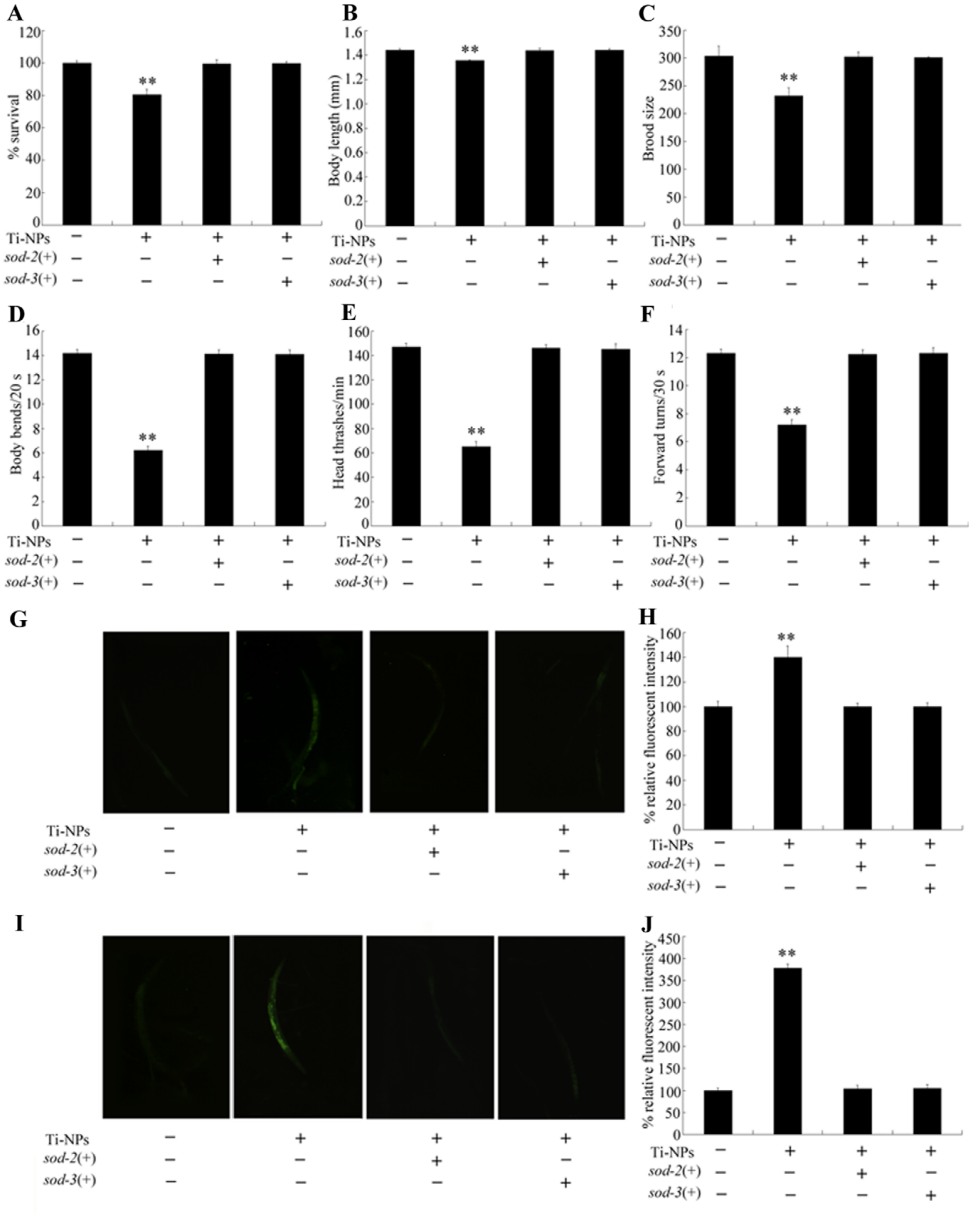
Effects of ectopically expression of nematode *sod-2* or *sod-3* gene on the toxicity formation in animals exposed to 4 nm TiO_2_-NPs at the concentration of 10 µg/L. (A) Effects of ectopically expression of *sod-2* or *sod-3* gene on the survival of nematodes exposed to 4 nm TiO_2_-NPs at the concentration of 10 µg/L. (B) Effects of ectopically expression of *sod-2* or *sod-3* gene on the growth of nematodes exposed to 4 nm TiO_2_-NPs at the concentration of 10 µg/L. (C) Effects of ectopically expression of *sod-2* or *sod-3* gene on the reproduction of nematodes exposed to 4 nm TiO_2_-NPs at the concentration of 10 µg/L. (D–F) Effects of ectopically expression of *sod-2* or *sod-3* gene on the locomotion behavior of nematodes exposed to 4 nm TiO_2_-NPs at the concentration of 10 µg/L. (G–H) Effects of ectopically expression of *sod-2* or *sod-3* gene on the intestinal autofluorescences of nematodes exposed to 4 nm TiO_2_-NPs at the concentration of 10 µg/L. (I–J) Effects of ectopically expression of *sod-2* or *sod-3* gene on the ROS production of nematodes exposed to 4 nm TiO_2_-NPs at the concentration of 10 µg/L. *sod-2*(+), *Ex*(P*sod-2-sod-2*); *sod-3*(+), *Ex*(P*sod-3-sod-3*). Exposure of TiO_2_-NPs was performed from L1-larvae, and the endpoints were examined when nematodes developed into the adults. Ti-NPs, TiO_2_-NPs. The Bars represent mean±S.E.M. **p<0.01.

To confirm the important roles of *sod-2* and *sod-3* genes in regulation of the formation of toxicity in nematodes exposed to different diameters of TiO_2_-NPs, we investigated the effects of ectopically expression of *sod-2* and *sod-3* genes upon toxicity development in nematodes exposed to 4-nm TiO_2_-NPs at 10 µg/L. Ectopically expression of *sod-2* or *sod-3* genes effectively prevented the increase in mortality, decrease in body length, reduction of brood size, decrease in locomotion behavior (as reflected by body bend, head thrash and forward turn), increase of intestinal autofluorescence, and induction of significant production of ROS formed in nematodes exposed to 4-nm TiO_2_-NPs at 10 µg/L (Fig. 3).

### Ectopically expression of human *SOD2* prevents the toxicity formation in nematodes exposed to TiO_2_-NPs

**Figure 4:**
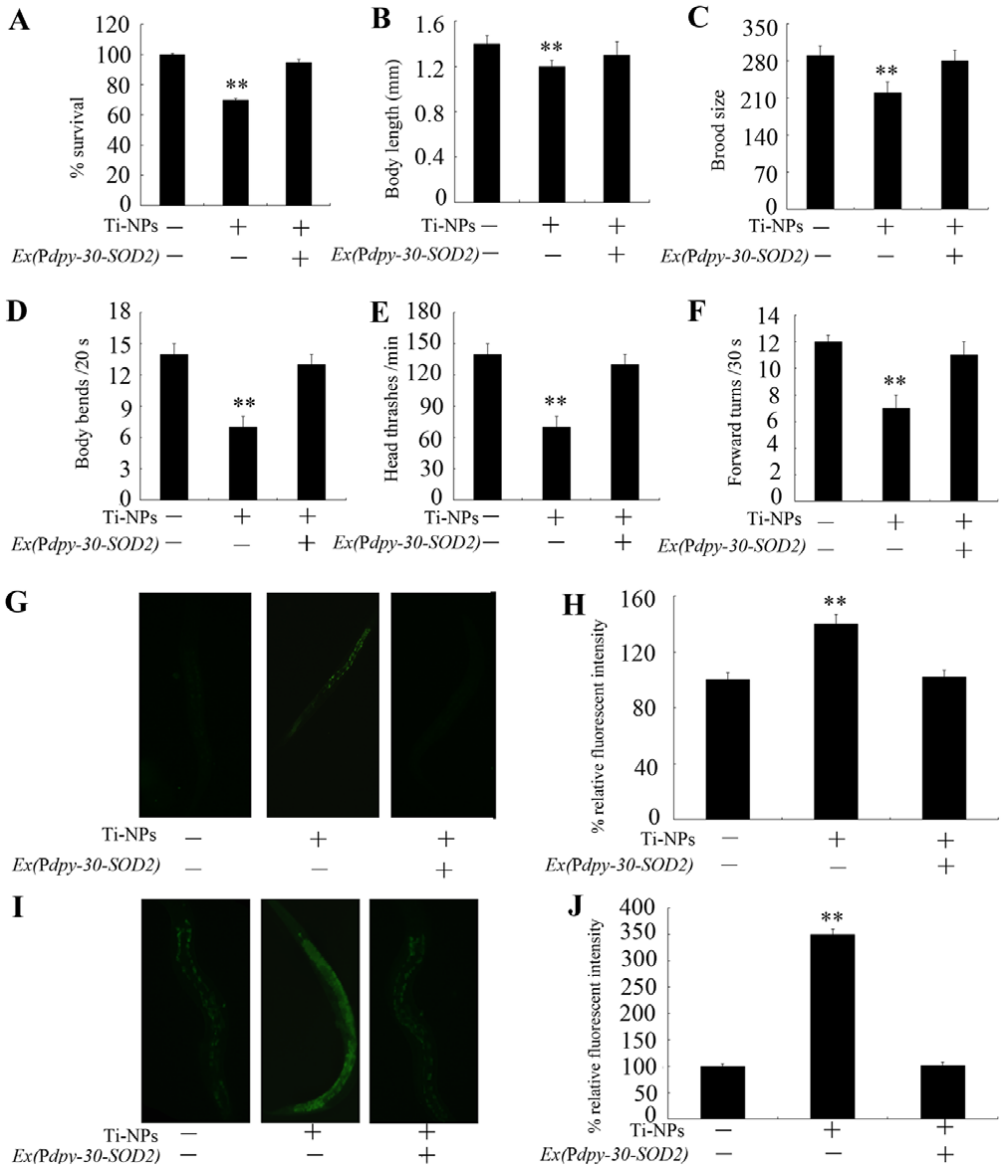
Effects of ectopically expression of human *SOD2* gene on the toxicity formation in animals exposed to 4 nm TiO_2_-NPs at the concentration of 10 µg/L. (A) Effects of ectopically expression of human *SOD2* gene on the survival of animals exposed to 4 nm TiO_2_-NPs at the concentration of 10 µg/L. (B) Effects of ectopically expression of human *SOD2* gene on the growth of animals exposed to 4 nm TiO_2_-NPs at the concentration of 10 µg/L. (C) Effects of ectopically expression of human *SOD2* gene on the reproduction of animals exposed to 4 nm TiO_2_-NPs at the concentration of 10 µg/L. (D–F) Effects of ectopically expression of human *SOD2* gene on the locomotion behavior of animals exposed to 4 nm TiO_2_-NPs at the concentration of 10 µg/L. (G–H) Effects of ectopically expression of human *SOD2* gene on the intestinal autofluorescences of animals exposed to 4 nm TiO_2_-NPs at the concentration of 10 µg/L. (I–J) Effects of ectopically expression of human *SOD2* gene on the ROS production of animals exposed to 4 nm TiO_2_-NPs at the concentration of 10 µg/L. Exposure of Ti-NPs was performed from L1-larvae, and the endpoints were examined when nematodes developed into the adults. Ti-NPs, TiO_2_-NPs. The Bars represent mean±S.E.M. **p<0.01.

In humans, the *SOD2* gene encodes the Mn-SODs. SOD-2 and SOD-3 in nematodes are highly homologous with SOD2 in humans (Fig. S1). With the aid of the *dpy-30* gene promoter to drive the human *SOD2* gene to express in all the cells of nematodes [54], we found that ectopically expression of the human *SOD2* gene also effectively suppressed the increase in mortality, decrease in body length, reduction of brood size, decrease in locomotion behavior (as reflected by body bend, head thrash and forward turn), increase of intestinal autofluorescence, and induction of significant production of ROS formed in nematodes exposed to 4-nm TiO_2_-NPs at 10 µg/L (Fig. 4).

## Discussion

Studies have shown that acute exposure to 50-nm TiO_2_-NPs at >100 mg/L causes a significant increase in mortality, and that the median lethal concentration (LC_50_) of nematodes acutely exposed to 25-nm TiO_2_-NPs was 77 mg/L [19], [37]. Acute exposure to TiO_2_-NPs (50 nm) at >47.9 mg/L resulted in a significant inhibition of growth, and a reduction of the reproduction of *C. elegans* [37]. In the present study, after exposure from L1-larvae to adulthood, a significant increase in intestinal autofluorescence was observed in nematodes exposed to 4-nm and 10-nm TiO_2_-NPs at the concentration of 1 µg/L. A significant increase in mortality, reduction in body length, and decrease of forward turns could be observed in nematodes exposed to 4-nm and 10-nm TiO_2_-NPs at the concentration of 0.1 µg/L. A significant reduction in brood size and induction of ROS production could be detected in nematodes exposed to 4-nm and 10-nm TiO_2_-NPs at the concentration of 0.01 µg/L. In particular, significant decreases in body bend and head thrash could be observed in nematodes exposed to 4-nm and 10-nm TiO_2_-NPs at the concentration of 0.001 µg/L (Figs 1–2). The limit of detection for most methods is not sufficiently low to detect environmentally relevant concentrations of engineered NPs in the range of ng/L to pg/L [55]. The predicted environmental concentrations in water for TiO_2_-NPs are 16 or 24.5 µg/L [56]–[57]. Therefore, using *C. elegans* as a bio-indicator, we can effectively assess the possible adverse effects of TiO_2_-NPs exposure at predicted environmental relevant concentrations on animals. Among the examined endpoints, body bend and head thrash are very sensitive, and can be used to evaluate the potential toxicity of TiO_2_-NPs exposure at the concentration of 1 ng/L. Similarly, after exposure from L1-larvae to adulthood, nano-CeO_2_ exhibited adverse effects on nematodes at environmental relevant concentrations [35].

In the present study, we compared the possible differences in toxicities of two groups of TiO_2_-NPs in nematodes. The first group comprised 4-nm and 10-nm TiO_2_-NPs (small diameters of TiO_2_-NPs), and the second group comprised 60-nm and 90-nm TiO_2_-NPs (large diameters of TiO_2_-NPs). When we detected the toxicity of small-size TiO_2_-NPs at relatively low concentrations, the large size of TiO_2_-NPs did not usually exhibit toxicity in nematodes. For example, when we observed a significant increase in mortality, reduction in body length, and decrease of forward turns in nematodes exposed to 4-nm and 10-nm TiO_2_-NPs at 0.1 µg/L, no noticeable alterations in lethality, body length and forward turns were found in nematodes exposed to 60-nm and 90-nm TiO_2_-NPs at 0.1 µg/L, and the lethality and body length of nematodes exposed to 1–10 µg/L of 60-nm and 90-nm TiO_2_-NPs were also not significantly affected (Figs 1A, 1B, and 1F). Moreover, when we detected a significant reduction in brood size and induction of ROS production in nematodes exposed to 4-nm and 10-nm TiO_2_-NPs at 0.01–0.1 µg/L, no obvious alterations in brood size and ROS production were found in nematodes exposed to 60-nm and 90-nm TiO_2_-NPs at 0.01–0.1 µg/L (Fig 1C, Figs 2A and 2B). In particular, when we observed the significant decreases in body bend and head thrash in nematodes exposed to 4-nm and 10-nm TiO_2_-NPs at 0.001–0.01 µg/L, the body bends and head thrashes in nematodes exposed to 60-nm and 90-nm TiO_2_-NPs at 0.001–0.01 µg/L were not significantly affected (Fig. 1D and 1E). Therefore, toxicity differences can be formed for different sizes of TiO_2_-NPs in nematodes exposed to predicted environmental relevant concentrations. These data are consistent with observations on the differences in the acute toxicity of TiO_2_-NPs with different nano levels in *C. elegans* [19]–[20]. Our data are also in agreement with the observations on the toxicity differences of TiO_2_-NPs with different nano levels in zebrafish and mice [11], [18].

ROS production was closely correlated with lethality, growth, reproduction, locomotion behavior and intestinal autofluorescence in nematodes exposed to different diameters of TiO_2_-NPs at predicted environmentally relevant concentrations (Table S1). Hence, we investigated the possible molecular basis of oxidative stress in regulating the formation of toxicity differences in nematodes exposed to different diameters of TiO_2_-NPs. Interestingly, exposure to TiO_2_-NPs only noticeably altered the expression patterns of *sod-2* and *sod-3* genes among the examined genes controlling the formation of oxidative stress in nematodes (Fig. 2C). In *C. elegans*, *sod-2* and *sod-3* genes encode Mn-SODs [48]. The expression levels of *sod-2* and *sod-3* also increased with the gradual increase in exposure concentrations of the examined TiO_2_-NPs (Figs 2D and 2E). Moreover, the linear regression analysis indicated that *sod-2* gene expression and *sod-3* expression were closely correlated with lethality, growth, reproduction, locomotion behavior, intestinal autofluorescence, and ROS production in nematodes exposed to all the examined different diameters of TiO_2_-NPs at predicted environmental relevant concentrations (Table S2). These data suggested that the specific expression patterns of Mn-SODs may explain the toxicity differences of Ti-NPs at different nano levels in animals. That is, the formation of toxicity differences from exposures to different sizes of TiO_2_-NPs may be primarily due to the formation of different expression levels of Mn-SODs in nematodes exposed to different sizes of TiO_2_-NPs. Nevertheless, we cannot exclude the possibility that specific gene expression patterns may form in nematodes exposed to different diameters of TiO_2_-NPs. For example, exposure to 7-nm TiO_2_-NPs and exposure to 20-nm TiO_2_-NPs have been demonstrated to exhibit different expression patterns of gene *cyp35a2*, which encodes a xenobiotic metabolism enzyme in nematodes [20]. Analyses of the expression of an entire genome revealed the differential effects of TiO_2_ nanotubes on vascular cells [58]. Analyses of the expression of an entire genome may help us to further reveal the possible specific gene expression patterns formed in nematodes exposed to TiO_2_-NPs at different nano levels in *C. elegans*. Increasing evidence has suggested that TiO_2_-NPs toxicity is dependent not only upon size, but also varies with particle shape, surface coating and functionalization [59]. Therefore, the other possible molecular mechanisms explaining the influences of particle shape, surface coating and functionalization on the formation of TiO_2_-NPs toxicity need the further elucidation.

Previous *in vivo* toxicity assays on TiO_2_-NPs suggested that subchronic dermal exposure to TiO_2_-NPs resulted in the significant changes in levels of SOD and MDA in the skin and liver tissues of mice, implying that pathological lesions may be mediated through the oxidative stress induced by deposited TiO_2_-NPs [11]. Similarly, alterations in the levels of biomarkers of oxidative stress suggested the formation of oxidative stress in the liver and gut tissues of zebrafish or the brains of rainbow trout [18], [21]. Nevertheless, no direct evidence has been provided to prove the important role of oxidative stress in inducing the formation of *in vivo* toxicity by TiO_2_-NPs. Our data indicated that ectopically expression of the human *SOD2* gene and nematode *sod-2* and *sod-3* genes not only prevented the induction of ROS production but also prevented the formation of toxicity in nematodes as assessed by the endpoints of lethality, growth, reproduction, locomotion behavior and intestinal autofluorescence in nematodes exposed to TiO_2_-NPs (Figs 3 and 4). These findings provided direct evidence of the key role of oxidative stress in inducing the *in vivo* toxicity of TiO_2_-NPs. In humans, *SOD2* encodes Mn-SODs, a major mitochondrial antioxidative enzyme that constitutes an important “control switch” in the generation of oxidative signals [60]. In the present study, we investigated the toxicological effects of exposure to NPs on humans by generating transgenic strains in nematodes for specific human genes.

In summary, the present study showed that toxicity differences formed in nematodes exposed to different diameters of TiO_2_-NPs at predicted environmental relevant concentrations when using lethality, growth, reproduction, locomotion behavior, intestinal autofluorescence, and ROS production as endpoints. Our data also imply that the intestine, neuron, and reproductive organs may serve as the target organs for TiO_2_-NPs in nematodes. Among the examined endpoints, ROS production was closely correlated with other endpoints in nematodes exposed to TiO_2_-NPs. Exposure to TiO_2_-NPs altered the expression patterns of only *sod-2* and *sod-3* genes (which encode the Mn-SODs) among the examined genes regulating oxidative stress in nematodes. *sod-2* gene expression and *sod-3* gene expression were closely correlated with lethality, growth, reproduction, locomotion behavior, intestinal autofluorescence, and ROS production in nematodes exposed to TiO_2_-NPs. In particular, ectopically expression of human and nematode Mn-SODs prevented the induction of ROS production and the toxicity formation in nematodes exposed to TiO_2_-NPs. Therefore, our data reveal the possible molecular basis for oxidative stress in regulation of the formation of toxicity differences from different sizes of TiO_2_-NPs at predicted environmental relevant concentrations.

## Materials and Methods

### Reagents and preparation of TiO_2_-NPs suspensions

Nanosized TiO_2_-NPs powders (4 nm and 10 nm; Zhejiang Wanjin Material Technology Co., Ltd, Zhejiang, China; 60 nm and 90 nm, Zhejiang Hongsheng Material Technology Co., Ltd, Zhejiang, China) were used without coating. The purities of these TiO_2_-NPs were 99.5% (4 nm), 99.5% (10 nm), 99.6% (60 nm), and 99.5% (90 nm). The particle diameters of these TiO_2_-NPs were 4±1 nm (4 nm), 10±1 nm (10 nm), 60±9 nm (60 nm), and 90±10 nm (90 nm). The surface properties of these TiO_2_-NPs were hydrophobic. The surface area of these TiO_2_-NPs was 200 m^2^/g (4 nm), 160 m^2^/g (10 nm), 41 m^2^/g (60 nm), and 40 m^2^/g (90 nm) based on measurement by N_2_ sorption at 77 K using a NOVA 1000e Surface Area Analyzer (Nova, Boynton Beach, FL, USA). The prepared stock suspension concentrations of TiO_2_-NPs were 0.001, 0.01, 0.1, 1, and 10 µg/L. A series of stock suspensions of TiO_2_-NPs was dispersed in K-medium (32 mM KCl, 51 mM NaCl) [23] by probe sonication at 100 W and 40 kHz for 30 min to form the suspensions used. During testing periods, the suspension of TiO_2_-NPs was stable and uniform in the K-medium throughout the experimental period, which was confirmed by observations under the microscope. All the other chemicals were obtained from Sigma–Aldrich (St. Louis, MO, USA).

### Strain preparation

The nematodes used in the present study were wild-type N2, originally obtained from the *Caenorhabditis* Genetics Center (funded by the NIH National Center for Research Resource, Bethesda, MD, USA) and transgenic strains of *Ex*(P*sod-2-sod-2*), *Ex*(P*sod-3-sod-3*), and *Ex*(P*dpy-30-SOD2*), which were maintained on nematode growth medium (NGM) plates seeded with *Escherichia coli* OP50 at 20°C as described [61]. Gravid nematodes were washed off the plates into centrifuge tubes, and were lysed with a bleaching mixture (0.45 M NaOH, 2% HOCl). Age-synchronized populations of L1-larval nematodes were obtained by the collection as described [24]. Exposures to different nano-sizes of TiO_2_-NPs at 0.001, 0.01, 0.1, 1, and 10 µg/L were done from the L1-larval stage in 12-well sterile tissue culture plates in a 20°C incubator in the presence of food. The nematodes were used for toxicity evaluation using lethality, growth, locomotion behavior, reproduction, intestinal autofluorescence, and ROS production as endpoints and for the gene expression pattern analysis when they developed into young adults.

### Lethality

A 1.0-mL aliquot of test solution for TiO_2_-NPs was added to each well of the tissue culture plates, which was subsequently loaded with 50 nematodes for each concentration. Fifty nematodes were loaded in each well, and three replicated were performed. After exposure, the inactive ones were scored under a dissecting microscope. Nematodes were judged to be dead if they did not respond to a stimulus using a small, metal wire. Lethality was evaluated by the percentage of surviving animals.

### Locomotion behavior

Locomotion behavior was assessed by the endpoints of head thrash, body bend and forward turn [52]. To assess head thrash, each examined nematode was transferred into a microtiter well containing 60 µL of modified K-medium on the top of agar. Head thrashes were counted for 1 min after a 1-min recovery period. A thrash was defined as a change in the direction of bending at the middle of the body. To evaluate the body bend, nematodes were placed onto a second plate and the number of body bends over 20 s was recorded. A body bend was considered to be a change in the direction of the part of the nematode corresponding to the posterior bulb of the pharynx along the *y* axis assuming that the nematode was traveling along the *x* axis. To evaluate a forward turn, forward sinusoidal movement in a 30-s interval was measured. Fifty nematodes were examined per treatment.

### Reproduction

After exposure in 1.0-mL aliquot of test solution for TiO_2_-NPs, reproduction was assessed by brood size of adult nematodes. To assess the brood size, we counted the number of offspring at all stages after the egg stage. Twenty replicates were examined per treatment.

### Growth

After exposure in 1.0-mL aliquot of test solution for TiO_2_-NPs, growth was assessed by body length. Body length was determined by measuring the flat surface area of young adult nematodes using Image-Pro® Express software. Twenty replicates were examined per treatment.

### Intestinal autofluorescence

Intestinal autofluorescence is caused by lysosomal deposits of lipofuscin, which can accumulate over time in aging nematodes [53]. Images were collected for fluorescence in the endogenous intestine using a 525-nm bandpass filter and without automatic gain control to preserve the relative intensity of the fluorescence of different animals. Nematodes were photographed on the same day to avoid the effects of variance in light sources on fluorescence intensity. Fluorescence was recorded and color images taken for the documentation of results with Magnafire® software (Olympus, Irving, TX, USA). Lipofuscin levels were measured using ImageJ Software (NIH Image, Bethesda, MD, USA) by determining the mean pixel intensity in the intestine of each animal. Twenty replicates were examined per treatment.

### ROS production

To ascertain if TiO_2_-NPs treatment activated oxidative damage, ROS production was assayed. Nematodes were transferred to M9 buffer containing 1 µM of 5-(and-6)-chloromethyl-2′,7′-dichlorodihydrofluorescein diacetate, acetyl ester (CM-H2DCFDA) to pre-incubate for 3 h at 20°C, and then mounted on agar pads for examination with a Laser Scanning Confocal Microscope (Leica, TCS SP2, Bensheim, Germany) at an excitation wavelength of 488 nm and emission wavelength of 510 nm. The relative fluorescence intensities of the intestines were semi-quantified. The semi-quantified ROS were expressed as relative fluorescent units (RFU). Twenty replicates were examined per treatment.

### Reverse transcription-polymerase chain reaction

Total RNA of nematodes was extracted using RNeasy Mini Kit (Qiagen, Valencia, CA, USA). Total RNA was reverse-transcribed using cDNA Synthesis kit (Bio-Rad Laboratories, Hercules, CA , USA), and real-time PCR was performed using primers for target genes of *clk-1* (forward primer, 5′-CACATACTGCTGCTTCTCGT-3′; reverse primer, 5′-TGAACCAACAGATGAACCTT-3′), *clk-2* (forward primer, 5′-TATCCTTTGTTGGTTTTGCC-3′; reverse primer, 5′-CAAATACACTCTACACCGCA-3′), *ctl-1* (forward primer, 5′-CTCCTACACGGACACGCAT-3′; reverse primer, 5′-GCATCTCCCTGGCTTTCAT-3′), *ctl-2* (forward primer, 5′-CGAACAGCTTCAACTATGG-3′; reverse primer, 5′-GTGGCTGGGAATGTGGTAT-3′), *ctl-3* (forward primer, 5′-TTCTCCTACACGGACACGC-3′; reverse primer, 5′-GCATCTCCCTGGCTTTCAT-3′), *gas-1* (forward primer, 5′-CTTGGTCTTTGGCTGTTGA-3′; reverse primer, 5′-CTTGGTCTTTGGCTGTTGA-3′), *isp-1* (forward primer, 5′-GCAGAAAGATGAATGGTCC-3′; reverse primer, 5′-CAGAAGCGTCGTAGTGAGA-3′), *mev-1* (forward primer, 5′-GGAATTCGCTTCTTAGGAT-3′; reverse primer, 5′-GCAGTCTTGTTGCTCTTGT-3′), *sod-1* (forward primer, 5′-ACGCTCGTCACGCTTTAC-3′; reverse primer, 5′-TCTTCTGCCTTGTCTCCG-3′), *sod-2* (forward primer, 5′-GGCATCAACTGTCGCTGT-3′; reverse primer, 5′-ACAAGTCCAGTTGTTGCC-3′) and *sod-3* (forward primer, 5′-TGACATCACTATTGCGGT-3′; reverse primer, 5′-GGGACCATTCCTTCCAAA-3′), *sod-4* (forward primer, 5′-CACCAGATGACTCGAACA-3′; reverse primer, 5′-AATGAGGCAAGAGAGTCG-3′), *sod-5* (forward primer, 5′-ATATTGCCAATGCCGTTC-3′; reverse primer, 5′-CTCTTCACCTTCGGCTTT-3′), *SOD2* (forward primer, 5′-AGGAACGGGGACACTTAC-3′; reverse primer, 5′-GAAGGTAGTAAGCGTGCT-3′), and reference gene of *act-1* (forward primer, 5′-CTGCAGATGTGTGACGACGAGGTT-3′; reverse primer, 5′-CTGCAGGAAGCACTTGCGGTGAAC-3′). Relative quantification of target genes in comparison to reference *act-1* gene was determined, and the final results were expressed as relative expression ratio (between target gene and reference gene) in the treatments as compared to the ratio in the control. Five replicates were performed per treatment.

### DNA construct and germline transformation

The *sod-2* (1166 bp, HindIII/SmaI) or *sod-3* (1869 bp, BamHI/SmaI) gene promoter fragment was subcloned into the pPD95_75 vector, and the *sod-2* or *sod-3* full length cDNA was inserted into the site of SmaI/KpnI of the pPD95_75 vector behind P*sod-2* or P*sod-3* fragment to obtain plasmid of P*sod-2-sod-2* or P*sod-3-sod-3*. To construct the plasmid of P*dpy-30-SOD2*, *dpy-30* gene promoter fragment (1907 bp, PstI/BamHI) was subcloned into the pPD95_75 vector, and the human *SOD2* full length cDNA was inserted into the site of SmaI/KpnI of the pPD95_75 vector behind P*dpy-30* fragment. Transgenic nematodes of *Ex*(P*sod-2-sod-2*), *Ex*(P*sod-3-sod-3*), and *Ex*(P*dpy-30-SOD2*) were generated as described [62]. The plasmids were injected as a mix at 20 ng/µl using P*dop-1::rfp* as a transgenic marker. Ectopically expressions of nematodes genes of *sod-2* and *sod-3* and human *SOD2* gene were confirmed by real-time PCR (Fig S2).

### Statistical analysis

All data were expressed as means±standard error of the mean (S.E.M.). Statistical analysis was performed using SPSS 12.0 (SPSS Inc., Chicago, IL, USA). Analysis of variance (ANOVA) was used to determine the significance of differences between the groups. Probability levels of 0.05 and 0.01 were considered statistically significant. Associations of ROS production and gene expression with other endpoints were assessed with linear regression analysis. Correlations between *sod-2* or *sod-3* gene expression and ROS production were also analyzed by Spearman's rank correlation.

## Supporting Information

Figure S1 **Protein sequence alignment between human SOD2 protein with SOD-2 and SOD-3 proteins in** ***C. elegans*** **.** “*” indicate the positions which have a single, fully conserved residue. The results showed that the protein sequences' identity between human SOD2 and *C. elegans* SOD-2 is 64.07%, and the identity between human SOD2 and *C. elegans* SOD-3 is 61.04%.
(DOC)

Figure S2 **Confirmation of the ectopically expression of nematode** ***sod-2*** **or** ***sod-3*** **gene** **and human** ***SOD2*** **gene in wild-type N2 nematodes.** Relative expression ratios (between target genes and *act-1* reference gene) in transgenic strains were normalized to the wild-type N2.
(DOC)

Table S1 **Associations of ROS production with lethality, growth, reproduction, locomotion behavior and intestinal autofluorescence in nematodes exposed to TiO_2_-NPs as assayed by linear regression analysis.**
(DOC)

Table S2 **Associations of** ***sod-2*** **or** ***sod-3*** **gene expression with lethality, growth, reproduction, locomotion behavior, intestinal autofluorescence, and ROS production in nematodes exposed to TiO_2_-NPs as assayed by linear regression analysis.**
(DOC)

Table S3 **Sperman's rank correlation coefficients (r) between** ***sod-2*** **or** ***sod-3*** **gene expression and ROS production in TiO_2_-NPs exposed nematodes.** *p<0.05; **p<0.01.
(DOC)
