# Supplementary material for: Molecular Control of TiO2-NPs Toxicity Formation at Predicted Environmental Relevant Concentrations by Mn-SODs Proteins
Source: PLoS One. 2012 Sep 4;7(9):e44688. doi: 10.1371/journal.pone.0044688 (PMC3433426; doi:10.1371/journal.pone.0044688)
Supplement: Figure S1 — Protein sequence alignment between human SOD2 protein with SOD-2 and SOD-3 proteins in C. elegans . “*” indicate the positions which have a single, fully conserved residue. The results showed that the protein sequences' identity between human SOD2 and C. elegans SOD-2 is 64.07%, and the identity between human SOD2 and C. elegans SOD-3 is 61.04%. (DOC) [file pone.0044688.s001.doc]

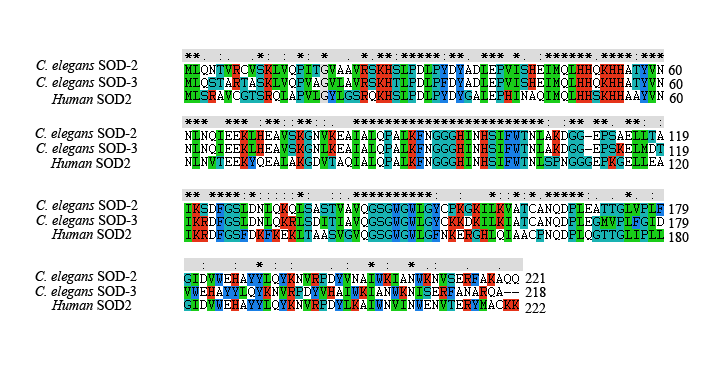


**Figure S1. Protein sequence alignment between human SOD2 protein with SOD-2 and SOD-3 proteins in *C. elegans*.**  “*” indicate the positions which have a single, fully conserved residue. The results showed that the protein sequences’ identity between human SOD2 and *C. elegans* SOD-2 is 64.07%, and the identity between human SOD2 and *C. elegans* SOD-3 is 61.04%.
