# Supplementary material for: Molecular Control of TiO2-NPs Toxicity Formation at Predicted Environmental Relevant Concentrations by Mn-SODs Proteins
Source: PLoS One. 2012 Sep 4;7(9):e44688. doi: 10.1371/journal.pone.0044688 (PMC3433426; doi:10.1371/journal.pone.0044688)
Supplement: Figure S2 — Confirmation of the ectopically expression of nematode sod-2 or sod-3 gene and human SOD2 gene in wild-type N2 nematodes. Relative expression ratios (between target genes and act-1 reference gene) in transgenic strains were normalized to the wild-type N2. (DOC) [file pone.0044688.s002.doc]

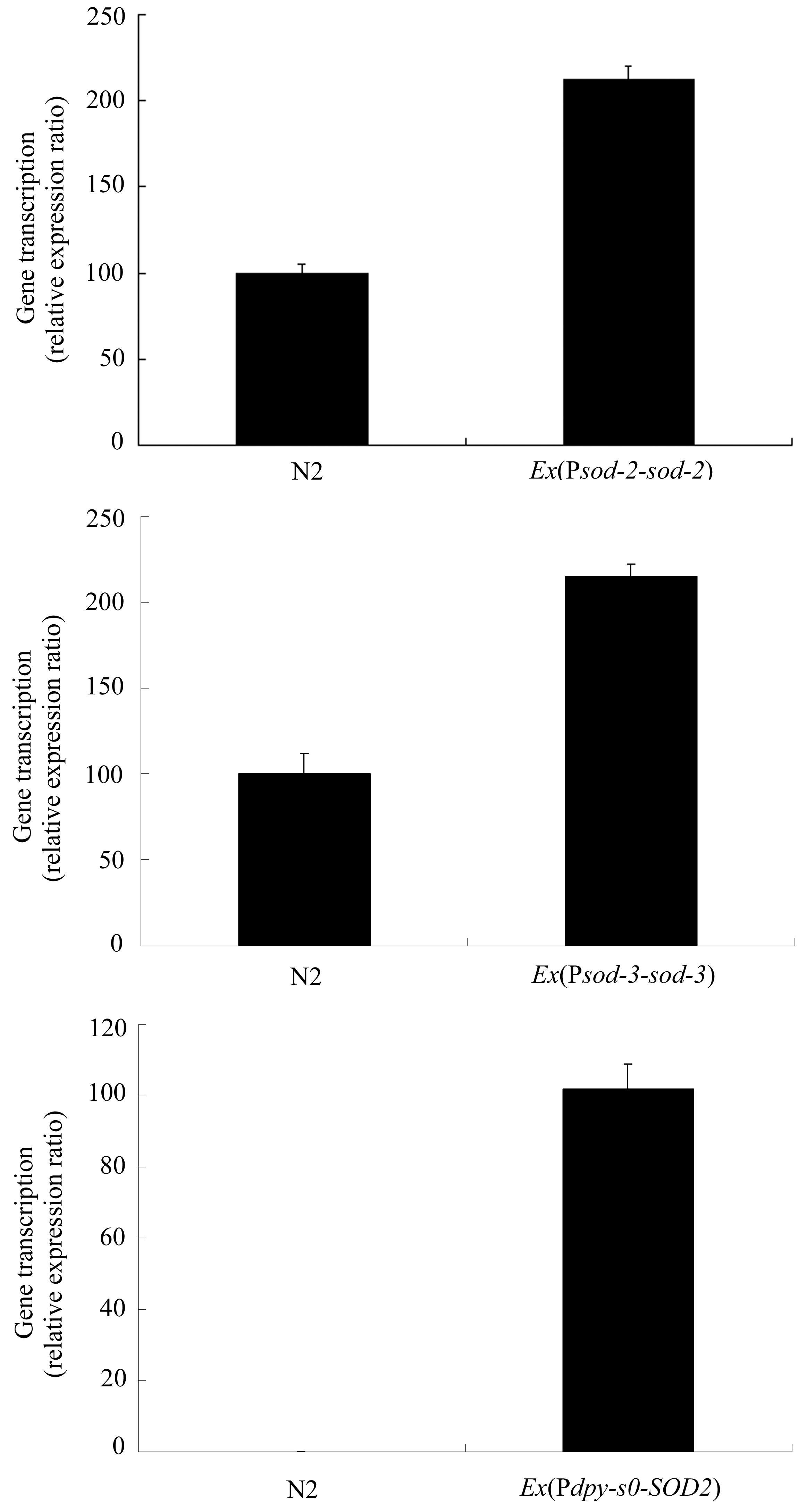


**Figure S2. Confirmation of the ectopically expression of nematode *sod-2* or *sod-3* gene** **and human *SOD2* gene in wild-type N2 nematodes.** Relative expression ratios (between target genes and *act-1* reference gene) in transgenic strains were normalized to the wild-type N2.
