# Supplementary material for: Molecular Control of TiO2-NPs Toxicity Formation at Predicted Environmental Relevant Concentrations by Mn-SODs Proteins
Source: PLoS One. 2012 Sep 4;7(9):e44688. doi: 10.1371/journal.pone.0044688 (PMC3433426; doi:10.1371/journal.pone.0044688)
Supplement: Table S1 — Associations of ROS production with lethality, growth, reproduction, locomotion behavior and intestinal autofluorescence in nematodes exposed to TiO2-NPs as assayed by linear regression analysis. (DOC) [file pone.0044688.s003.doc]

**Table S1 Associations of ROS production with lethality, growth, reproduction, locomotion behavior and intestinal autofluorescence in nematodes exposed to TiO_2_-NPs as assayed by linear regression analysis.**

|  | Dependent variable | Independent variable | |
| --- | --- | --- | --- |
|  |  | ROS production | |
|  |  | *R^2^* | *P* value |
| Ti-NPs (4 nm) | Lethality | 0.985 | < 0.01 |
|  | Growth | 0.986 | < 0.01 |
|  | Reproduction | 0.828 | < 0.05 |
|  | Body bend | 0.935 | < 0.01 |
|  | Head thrash | 0.916 | < 0.01 |
|  | Forward turn | 0.996 | < 0.01 |
|  | Intestinal autofluorescence | 0.967 | < 0.01 |
| Ti-NPs (10 nm) | Lethality | 0.992 | < 0.01 |
|  | Growth | 0.985 | < 0.01 |
|  | Reproduction | 0.809 | < 0.05 |
|  | Body bend | 0.960 | < 0.01 |
|  | Head thrash | 0.949 | < 0.01 |
|  | Forward turn | 0.978 | < 0.01 |
|  | Intestinal autofluorescence | 0.918 | < 0.01 |
| Ti-NPs (60 nm) | Lethality | 0.944 | < 0.01 |
|  | Growth | 0.724 | < 0.05 |
|  | Reproduction | 0.868 | < 0.01 |
|  | Body bend | 0.837 | < 0.05 |
|  | Head thrash | 0.839 | < 0.05 |
|  | Forward turn | 0.992 | < 0.01 |
|  | Intestinal autofluorescence | 0.878 | < 0.01 |
| Ti-NPs (90 nm) | Lethality | 0.981 | < 0.01 |
|  | Growth | 0.719 | < 0.05 |
|  | Reproduction | 0.900 | < 0.01 |
|  | Body bend | 0.795 | < 0.05 |
|  | Head thrash | 0.837 | < 0.05 |
|  | Forward turn | 0.997 | < 0.01 |
|  | Intestinal autofluorescence | 0.904 | < 0.01 |
