# Supplementary material for: Molecular Control of TiO2-NPs Toxicity Formation at Predicted Environmental Relevant Concentrations by Mn-SODs Proteins
Source: PLoS One. 2012 Sep 4;7(9):e44688. doi: 10.1371/journal.pone.0044688 (PMC3433426; doi:10.1371/journal.pone.0044688)
Supplement: Table S2 — Associations of sod-2 or sod-3 gene expression with lethality, growth, reproduction, locomotion behavior, intestinal autofluorescence, and ROS production in nematodes exposed to TiO2-NPs as assayed by linear regression analysis. (DOC) [file pone.0044688.s004.doc]

**Table S2 Associations of *sod-2* or *sod-3* gene expression with lethality, growth, reproduction, locomotion behavior, intestinal autofluorescence, and ROS production in nematodes exposed to TiO_2_-NPs as assayed by linear regression analysis.**

|  | Dependent variable | Independent variable | | | |
| --- | --- | --- | --- | --- | --- |
|  |  | *sod-2* expression | | *sod-3* expression | |
|  |  | *R^2^* | *P* value | *R^2^* | *P* value |
| Ti-NPs (4 nm) | ROS production | 0.914 | < 0.01 | 0.745 | < 0.05 |
|  | Lethality | 0.860 | < 0.01 | 0.684 | < 0.05 |
|  | Growth | 0.921 | < 0.01 | 0.797 | < 0.05 |
|  | Reproduction | 0.973 | < 0.01 | 0.918 | < 0.01 |
|  | Body bend | 0.985 | < 0.01 | 0.885 | < 0.01 |
|  | Head thrash | 0.973 | < 0.01 | 0.859 | < 0.01 |
|  | Forward turn | 0.887 | < 0.01 | 0.716 | < 0.05 |
|  | Intestinal autofluorescence | 0.823 | < 0.05 | 0.664 | < 0.05 |
| Ti-NPs (10 nm) | ROS production | 0.805 | < 0.05 | 0.807 | < 0.05 |
|  | Lethality | 0.772 | < 0.05 | 0.702 | < 0.05 |
|  | Growth | 0.810 | < 0.05 | 0.807 | < 0.05 |
|  | Reproduction | 0.997 | < 0.01 | 0.978 | < 0.01 |
|  | Body bend | 0.914 | < 0.01 | 0.905 | < 0.01 |
|  | Head thrash | 0.928 | < 0.01 | 0.914 | < 0.01 |
|  | Forward turn | 0.753 | < 0.05 | 0.754 | < 0.05 |
|  | Intestinal autofluorescence | 0.662 | < 0.05 | 0.668 | < 0.05 |
| Ti-NPs (60 nm) | ROS production | 0.823 | < 0.05 | 0.915 | < 0.01 |
|  | Lethality | 0.872 | < 0.01 | 0.959 | < 0.01 |
|  | Growth | 0.890 | < 0.01 | 0.786 | < 0.05 |
|  | Reproduction | 0.944 | < 0.01 | 0.985 | < 0.01 |
|  | Body bend | 0.864 | < 0.05 | 0.948 | < 0.01 |
|  | Head thrash | 0.859 | < 0.01 | 0.966 | < 0.01 |
|  | Forward turn | 0.696 | < 0.05 | 0.952 | < 0.01 |
|  | Intestinal autofluorescence | 0.681 | < 0.05 | 0.820 | < 0.05 |
| Ti-NPs (90 nm) | ROS production | 0.913 | < 0.01 | 0.956 | < 0.01 |
|  | Lethality | 0.966 | < 0.01 | 0.970 | < 0.01 |
|  | Growth | 0.874 | < 0.01 | 0.871 | < 0.01 |
|  | Reproduction | 0.982 | < 0.01 | 0.973 | < 0.01 |
|  | Body bend | 0.944 | < 0.01 | 0.867 | < 0.01 |
|  | Head thrash | 0.965 | < 0.01 | 0.918 | < 0.01 |
|  | Forward turn | 0.940 | < 0.01 | 0.975 | < 0.01 |
|  | Intestinal autofluorescence | 0.872 | < 0.01 | 0.875 | < 0.01 |
