# Supplementary material for: Molecular Control of TiO2-NPs Toxicity Formation at Predicted Environmental Relevant Concentrations by Mn-SODs Proteins
Source: PLoS One. 2012 Sep 4;7(9):e44688. doi: 10.1371/journal.pone.0044688 (PMC3433426; doi:10.1371/journal.pone.0044688)
Supplement: Table S3 — Sperman's rank correlation coefficients (r) between sod-2 or sod-3 gene expression and ROS production in TiO2-NPs exposed nematodes. *p<0.05; **p<0.01. (DOC) [file pone.0044688.s005.doc]

**Table S3 Sperman’s rank correlation coefficients (r) between *sod-2* or *sod-3* gene expression and ROS production in TiO_2_-NPs exposed nematodes.**  **p* < 0.05; ***p* < 0.01.

| ROS production | *sod-2* gene expression | *sod-3* gene expression |
| --- | --- | --- |
| Ti-NPs (4 nm) | 1.0^**^ | 1.0^**^ |
| Ti-NPs (10 nm) | 1.0^**^ | 1.0^**^ |
| Ti-NPs (60 nm) | 0.99^**^ | 0.98^**^ |
| Ti-NPs (90 nm) | 0.91^*^ | 0.9^*^ |
